# Sourdough Bread Quality: Facts and Factors

**DOI:** 10.3390/foods13132132

**Published:** 2024-07-04

**Authors:** Md Ahmadul Islam, Shahidul Islam

**Affiliations:** 1 Department of Plant Sciences, North Dakota State University, Fargo, ND 58108, USA; islam.mdahmadul@ndsu.edu; 2 Department of Food Technology and Rural Industries, Bangladesh Agricultural University, Mymensingh 2202, Bangladesh

**Keywords:** bread, fermentation, nutrition, particle size, protein, sourdough, starch

## Abstract

The term “sourdough” denotes a dough composed of flour and water, fermented through the action of yeast and lactic acid bacteria. The utilization of sourdough fermentation technology can enhance the nutritional attributes of bread made from wheat grain. In recent times, sourdough bread has experienced a resurgence, fueled by growing consumer demand for healthier bread options. The market dynamics for sourdough illustrate its rapid expansion and significant role in the contemporary food industry. Sourdough fermentation improves nutritional qualities by altering the structure and function of proteins and starch, enhancing dietary fiber, volatile compound profiles, and antioxidant activity, and reducing FODMAPs. The quality of sourdough bread is influenced by several factors, including fermentation environment, flour particle size, protein quality, starch characteristics, and dietary fiber composition. Moreover, the incorporation of alternative grains (intermediate wheatgrass and legume flour) and non-flour ingredients (fruits, herbs, and dairy products) presents opportunities for creating sourdough bread with unique sensory and nutritional profiles. This review offers updated insights on the quality aspects of sourdough fermentation, the factors that influence the effectiveness of the sourdough fermentation process, sourdough technology with unconventional and non-flour ingredients, and the potential market for frozen sourdough, considering its convenience and extended shelf life.

## 1. Introduction

Sourdough (SD) is one of the earliest technologies used to produce cereal-based foods [1]. The primary distinction between SD and the modern bread making formula is in the leavening process, specifically, the type of leavening agent used. Modern bread is typically made by using commercial yeast, a single strain (S. cerevisiae) of yeast added to the dough to help it rise. On the other hand, SD uses a natural leavening process that relies on natural yeasts and lactic acid bacteria (LAB). It is produced by the spontaneous fermentation of microbes found in flour or other raw materials [2,3], predominantly LAB. Table 1 represents commonly identified LAB and yeast in SD.

SD fermentation significantly improves bread quality in various ways. For instance, it slows down starch digestion, leading to a reduced glycemic response, enhances protein digestion, and boosts the absorption of minerals. Consuming SD bread also introduces more beneficial microbes and dietary fiber to the gut, enriching the diversity and quantity of intestinal bacteria and potentially benefiting human health [8,9]. SD fermentation also helps improve the taste and texture attributes of whole wheat bread [10,11]. Whole wheat flour (WWF) is typically more nutritious than refined flour. However, it reduces the sensory properties, which provide a dark color, speckled appearance, coarse and rugged texture, bitter/sour flavor, malted notes, and mustiness [12]. SD fermentation stands out as a popular method to improve the sensory characteristics of items made from WWF [11].

In recent decades, the SD market has demonstrated substantial growth in many regions, claiming a notable portion of the wheat-based baking industry. Nevertheless, SD fermentation is a complex procedure shaped by multiple factors that dictate the final product’s quality. Elements such as the kind and grade of flour, protein and starch properties, and fermentation method, including the duration, temperature, and technique of fermentation, all play roles in determining the quality of the SD product. Variations in the protein and starch properties of the flour change the SD bread texture and flavor. Optimizing those factors would provide the opportunity to make a high-quality sourdough product with the desired flavor, texture, and shelf life. This review summarizes the quality aspects of sourdough fermentation compared to yeast fermentation and the factors regulating the efficiency of the SD fermentation process.

## 2. SD Market Dynamics

With the growing global appetite for healthier baked goods, the SD market shows continued growth in the foreseeable future. The SD market is estimated to reach a valuation of USD 2.45 billion in 2024. It is projected to expand to USD 3.30 billion by 2029, marking a CAGR of 6.13% from 2024 to 2029 [13]. In developed countries such as the United States, Canada, the UK, and France, consumer awareness of SD benefits has been on the rise, bolstering the demand for SD products. On the other hand, in the Asia-Pacific region, the SD market has seen growth driven by changing consumer lifestyles, dietary choices, and the economic progress of countries such as India, Singapore, Australia, China, Japan, and South Korea. Between 2018 and 2028, the Asia Pacific recorded the fastest CAGR in the SD market [13].

The worldwide SD market spans a range of application segments. Predominantly, products like pizza bases, cakes, pastries, bread, and buns lead the global SD sector. Based on the fermentation method and the technological strategy employed, SD can be segmented into four categories. Type I, for instance, involves back-slopping methods stemming from a naturally fermented mix of flour and water and generally maintains a pH ranging from 3.8 to 4.5 [5,14]. Type I avoids the addition or inclusion of *S. cerevisiae* (baker’s yeast) in the form of a leaving agent. SD type II is recognized as an industrial approach since it involves a single fermentation stage of LAB solely or with yeast, lasts for 15–24 h, and is followed by back-slopping [5]. Starter cultures are added to the yeast in this type of SD at a ratio of 100:1. Industrial bakeries can simply pump this kind of formulation because it also comes in liquid form [5,14]. Products of type II can be stored or chilled for a week and have a pH value of 3.5 or less [14]. Type III is simply type II SD that has been dehydrated by spray drying or freeze drying [15]. The ability of the starter culture to quickly acidify the flour–water mixture and/or the production of particular flavors are the determining factors in starter culture selection [16]. However, baker’s yeast must be added to the dough to leaven it. Most businesses that make type III SD guarantee a stable starter culture so that it can be utilized as a sponge or leaven in the creation of bread after rehydrating. *P. pentosaceus, Lb. plantarum*, and *Lb. brevis* are a few examples of LAB that are resistant to drying [17]. Type IV is a laboratory-scale blend of type I and type II SD [5]. Contrasted with freshly prepared SD, type III sourdough proves to be more user-friendly and offers greater convenience in storage. This characteristic facilitates standardized industrial production and diminishes the requirement for maintaining SD starters. Therefore, type III SD has become the most widely used SD variety for commercial production [15]. Due to its better physical and chemical stability and less susceptibility to microbial contamination, dehydrated dough (type III) has a longer shelf life than fresh dough. These qualities have made SD more commercially viable and encouraged the production of various kinds of SD used as sponge dough starters. Table 2 shows the SD market in different nations, along with the flour that is used to make SD products.

## 3. SD Fermentation Compared to Yeast Fermentation

When comparing SD fermentation to yeast fermentation, notable differences arise in their outcomes (Table 3). These include variations in nutritional properties, health benefits, digestibility, protein and starch functionality, and the production of volatile compounds.

### 3.1. Improves Nutritional Properties and Human Health

**Figure 1:**
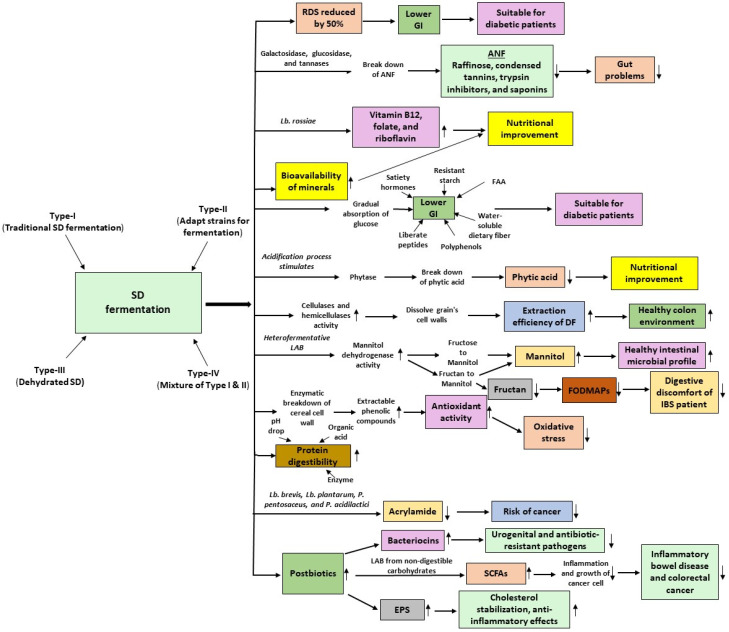
How SD fermentation influences the nutritional and health benefits of bread as compared to yeast fermentation. SD: sourdough; RDS: rapidly digestible starch; ANFs: anti-nutritional factors; DF: dietary fiber; GI: glycemic index; FAA: free amino acid; LAB: lactic acid bacteria; FODMAPs: fermentable oligosaccharides, disaccharides, monosaccharides, and polyols; SCFAs: short-chain fatty acids; EPS: exopolysaccharide. An upward arrow indicates an increase, while a downward arrow indicates a decrease.

SD fermentation offers several nutritional as well as health advantages over yeast fermentation in bread-making (Figure 1).

#### 3.1.1. Increasing Availability of Nutrients

SD demonstrated a higher potential to break down complex molecules into simpler forms during long fermentation. Some of the nutrient components, for example, minerals (calcium, salt, magnesium, iron, and zinc), might otherwise be retained inaccessible for digestion [39]. The total bioavailability of minerals such as iron and zinc in bread is enhanced by 10 and 25%, respectively, by SD fermentation [22,23,24,27]. In addition, *Lb. rossiae* DSM 15814 from SD contributes to nutritional value by boosting levels of vitamin B12, folate, and riboflavin [21]. SD fermentation also produces more fermentable sugars such as polyol, specifically sorbitol and mannitol (Table 4), which contribute to maintaining a healthy intestinal microbial profile [40].

#### 3.1.2. Reducing Phytate Content

Phytic acid, also known as myo-inositol hexaphosphate, is a natural constituent of grains. It forms insoluble complexes by binding to minerals and other bioactive compounds, which diminishes their dietary absorption. This can hinder the body’s ability to absorb essential minerals, particularly calcium, salt, magnesium, iron, and zinc [25]. SD fermentation can degrade phytic acid up to 96.6% when mixed fermentation is used to prepare type II SD [25,26]. Phytases are enzymes that break down phytic acid, subsequently releasing myo-inositol, smaller-sized inositol phosphate, and soluble inorganic phosphate. The acidification process in SD indirectly stimulates both the innate phytases in the flour and the microbial enzyme activity, resulting in a significant increase in mineral bioavailability [44]. *P. pentosaceus, Le. kimchi* VTT E-153484, *S. cerevisiae*, and *Wi. anomalus* P4 reduce phytate levels and enhance mineral solubilization in SD bread. On the other hand, yeast fermentation results in a reduction of up to 56% [25,26].

#### 3.1.3. Degradation of Anti-Nutritional Factors

Cereals contain anti-nutritional factors (ANFs), which can either restrict their consumption or result in serious illnesses. Raffinose, condensed tannins, vicine and convicine, saponins, and trypsin inhibitors are the primary ANFs in wheat flour [25], in addition to phytic acid (which is already described in Section 3.1.2.). Raffinose causes gut problems, which is not digestible by pancreatic enzymes but is fermentable by gas-producing bacteria in the large intestine. Additionally, proteins and other nutrients are poorly assimilated when digestive enzymes are inhibited by trypsin inhibitors and condensed tannins. Saponins, vicine, and convicine are examples of biologically active glycosides that hemolyze red blood cells and combine with nutrients to block absorption [25].

SD fermentation has the ability to break down ANFs [25]. A portfolio of enzymes, likely galactosidase, glucosidase, and tannases, are predominantly present in SD lactic acid bacteria in addition to lactic acidification and have the capacity to neutralize the presence of various ANFs. Vicine and con-vicine become completely destroyed by SD fermentation with specific *Lb. plantarum* within 48 h, and aglycone derivatives remain undetectable [25,45]. As a result of SD fermentation, the concentrations of raffinose (62–80%), condensed tannins (23–44%), trypsin inhibitors (23–44%), and saponins (68%) of WWF decrease [25,27]. The combination of gelatinization and SD fermentation significantly reduces the residual concentrations of condensed tannins (62%) and trypsin inhibitors (70%) in food [25,46]. Most of those activities do not take place in yeast fermentation.

#### 3.1.4. Probiotics and Postbiotics in SD: The Impact on Human Health

Probiotics and postbiotics play crucial roles in SD fermentation, significantly impacting human health compared to yeast fermentation. In SD, a symbiotic culture of LAB and wild yeasts creates an environment rich in probiotics [21]. Probiotics are live microorganisms that confer health benefits when consumed in adequate amounts. Probiotics enhance gut health by promoting a balanced microbiota, aiding digestion, and potentially bolstering the immune system. For example, *Lb. plantarum* ZJUFB2, derived from Chinese sourdough, exerts a probiotic effect on gut microbiota, aiding in the prevention of insulin resistance and the modulation of the gut microbiota; *Lb. plantarum* ZJUFT17 aids in regulating gut microbiota by decreasing pathogenic and proinflammatory microbes while encouraging the growth of anti-obesity bacteria [21]. It is important to note that most probiotics die when exposed to the high baking temperature. However, the health benefits remain, not through the probiotics colonizing intestinal epithelial cells but through the cells and metabolites produced during SD fermentation, such metabolites known as postbiotics [6]. Postbiotics are non-living microorganisms comprising inanimate microbial cells or their components that promote host health. The microbial metabolites and cellular structures from SD are potential sources of postbiotics [6]. Examples of postbiotic-like compounds present in SD include short-chain fatty acids (SCFAs), secreted proteins and peptides, bacteriocins, biosurfactants, amino acids, flavonoids, exopolysaccharides (EPSs), vitamins, organic acids, and a variety of other diverse molecules [6,21]. SCFAs, produced by LAB from non-digestible carbohydrates, aid in managing inflammatory bowel disease and colorectal cancer by reducing inflammation and inhibiting the growth of cancer cells [28]. EPSs, such as β-glucan, dextran, and inulin, are metabolites produced by LAB during SD fermentation. β-glucan, a glucose-based prebiotic homopolysaccharide, provides substantial health benefits, including cholesterol stabilization, anti-inflammatory effects, and support for probiotic microorganisms [21,28]. Bacteriocins have the potential to inhibit various urogenital and antibiotic-resistant pathogens [47]. Biosurfactants play a crucial role in disrupting and preventing biofilm formation by pathogenic microorganisms. They interfere with the wetting, foaming, and emulsification processes that pathogens rely on to adhere, establish themselves, and communicate within biofilms [6,28]. Peptidoglycan, a linear glycan strand cross-linked by peptides and composed of N-acetylglucosamine and N-acetylmuramic acid, has notable immunomodulatory, anti-proliferative, and anti-tumor properties [6]. In contrast, yeast fermentation, primarily utilizing commercial strains like *S. cerevisiae*, does not produce significant levels of probiotics or postbiotics [21]. While yeast fermentation is faster and more predictable, resulting in a uniform texture and volume in bread, it lacks the diverse microbial community and bioactive compounds characteristic of SD.

#### 3.1.5. Availability of Dietary Fiber

Compared to yeast fermentation, SD fermentation significantly increases dietary fiber availability [30]. SD fermentation boosts the activity of enzymes, such as cellulases and hemicellulases, which break down cell walls and improve the accessibility and extraction efficiency of dietary fiber. For example, *Lb. brevis* TMW 1.2112 and *P. claussenii* TMW 2.340 enhance dietary fiber availability, promoting a healthy colon environment and providing chemopreventive benefits [21]. Increasing the activity of protease while reducing the activity of amylase in SD makes the fiber more soluble [22,27]. SD fermentation, by activating xylanase, also facilitates the transformation of water-unextractable arabinoxylan (WUAX) in the bran into its water-extractable counterpart [11,22,27].

It is worth noting that certain types of dietary fiber components act as prebiotics after modification caused by SD fermentation. These prebiotics serve as a food source for beneficial gut bacteria. The enzymatic activity of LAB during fermentation contributes to the breakdown of arabinoxylan (AX), which yields arabinoxylan-oligosaccharides (AXOSs). AXOSs act as probiotics, even with a more robust efficacy than fructooligosaccharides [48]. These oligosaccharides stimulate the growth of beneficial bacteria in the gut [49] and potentially improve gut health and overall digestion [50]. However, the availability or solubility of dietary fiber is sensitive to the SD fermentation environment and the types of culture used.

#### 3.1.6. Increasing Resistant Starch

The term “resistant starch” describes the fraction of starch that avoids digestion in the small intestine and enters the large intestine, where intestinal bacteria can ferment [51]. Contrary to yeast fermentation, SD fermentation has been found to increase the amount of resistant starch in bread by 89% to 120% [29,30]. During the fermentation process of SD, LAB can lower the pH (from 6.5 to 3.5) of the dough through the production of lactic and acetic acid. This acidic environment can modify the structure of starch molecules, making them more resistant to digestion. In addition, LAB release enzymes such as α-amylase, β-xylosidase, and α-arabinofuranosidase that facilitate the conversion of starch into resistant starch varieties, including retrograde and inaccessible starch [30]. Thus, SD-fermented bread has higher quantities of resistant starch, which has several health advantages, including encouraging the development of good gut flora and enhancing digestive health [51]. Resistant starch also enhances the uptake of micronutrients and has a synergistic effect with other dietary components (dietary fibers, proteins, lipids). Specifically, it lowers postprandial insulin and glucose levels, increases the generation of colonic short-chain fatty acids, and decreases secondary bile acids, cecal ammonia, cecal bulking, and colonic transit time while maintaining high tolerance [51]. Additionally, it reduces pathogenic bacteria, increases beneficial colonic bifidobacteria and lactobacilli, and may interact beneficially with other probiotics like FOS and inulin. Furthermore, it boosts mineral absorption, especially calcium [51].

#### 3.1.7. Digestibility and Lowering the Glycemic Index (GI)

SD bread has superior digestibility compared to yeast bread. SD fermentation results in higher nutritional indices, faster gastric emptying, and a quicker oro-cecal transit time. Additionally, SD bread leads to lower postprandial glycemia and maintains higher levels of total free amino acids in blood plasma for a prolonged period [31].

The digestibility of starch, whether slowly digestible starch (SDS) or rapidly digestible starch (RDS), significantly influences glucose metabolism, diabetes management, and satiety. RDS triggers a high glycemic index (GI), making low RDS products more suitable for diabetic patients [32]. The GI gauges the rate at which food raises blood sugar levels. Foods with a low GI typically result in a steady, prolonged release of glucose, whereas high GI foods can trigger rapid spikes in blood sugar levels [52]. The Harvard Medical School classifies foods based on their GI into low (GI ≤ 55), moderate (GI between 55 and 69), and high (GI ≥ 70) categories [25].

Adding SD is an effective strategy for reducing RDS as well as GI. Compared to yeast fermentation, SD fermentation reduces the amount of RDS by approximately half [32]. SD fermentation has already been mentioned as leading to more gradual glucose absorption into the bloodstream, quicker gastric emptying, the activation of satiety hormones, and an increase in resistant starch [24,25,31]. It also liberates peptides, free amino acids, polyphenols, and water-soluble dietary fiber [31], all contributing to a lower GI. When 5 to 10% dietary fiber is incorporated and subjected to SD fermentation, the GI decreases to values under 55, categorizing these baked products as low GI foods suitable for various dietary preferences [25].

The organic acids produced by LAB, such as acetic acid and lactic acid from SD fermentation, aid in reducing the glycemic response. These acids inhibit starch-hydrolyzing enzymes, leading to slower starch digestion and a reduced GI [22,27].

#### 3.1.8. Reducing FODMAPs

FODMAPs are short-chain dietary carbohydrates, which stands for fermentable oligosaccharides, disaccharides, monosaccharides, and polyols. They can cause digestive discomfort in individuals, especially those with irritable bowel syndrome (IBS) or other gastrointestinal illnesses [53]. Compared to yeast fermentation, SD fermentation leads to a reduction of all FODMAPs, except polyols, in wheat bread [42,43,53,54,55]. Fructans make up the majority of the FODMAPs in wheat bread [54]. In wheat flour, the amount of fructans ranges from 1.4 to 1.7%, while in WWF, it ranges from 0.7 to 2.9% [55]. Thus, the breakdown of fructans is one of the most effective ways through which SD reduces the FODMAP content (Table 4). Type I SD typically contains heterofermentative bacteria, which has mannitol dehydrogenase activity that enables fructose to be used as an electron acceptor, converting it to mannitol [56]. Thus, mannitol is produced during SD fermentation from fructose and other fructose-containing sugars, including sucrose and fructans, boosting the quantities of sugar alcohol in the subsequent SD [56]. To create SD with low fructans and mannitol, a mix of *lactobacilli* that ferments fructans and mannitol would be required [56]. Fructans are also partially broken down in SD bread by fructan-degrading enzymes such as fructanase and inulinase, which are produced by homofermentative LAB belonging to genera such as *Lb. crispatus*, *Lb. delbrueckii*, *Lb. casei*, *Lb. plantarum*, and *Lb. salivarius* [55,56].

Strains of LAB vary in their capacity to break down fructans and lower FODMAPs in type II SD fermentation [42]. For example, *Lb. reuteri* 100-23 can decrease fructan content by only 13% after 16 h of SD fermentation, while *Lb. crispatus* DSM29598 in SD bread reduces fructan content by more than 90% and total FODMAPs by more than 70% [42]. Another study indicated that the consistent use of a pure culture of *Lb. plantarum* has a profound effect on decreasing the fructans in SD bread (up to 33%) [55].

The reduction level of FODMAPs in SD is also influenced by its acidity [35]. Increased acidity in SD increases the activities of several enzymes such as fructanase and inulinase, which break down many carbohydrates, including fructan, through many biochemical reactions, hence reducing FODMAPs [35]. Moreover, the longer time in fermentation (up to 72 h) in SD contributes to minimizing the FODMAP concentration [57]; however, the mechanism behind that has not been explored yet. Therefore, it would be valuable to examine how differences in bacterial strains and fermentation conditions might further decrease FODMAP production.

#### 3.1.9. Lowering Acrylamide

SD bread made from certain strains of LAB (*Lb. brevis S12, Lb. plantarum S28, P. pentosaceus S14,* and *P. acidilactici S16*) can effectively lower acrylamide levels [58]. Acrylamide, a carcinogenic compound formed during baking via the Maillard reaction, is mitigated by the low pH from SD fermentation, which hinders its synthesis [59]. One study found that SD bread made with various Lactobacillus strains (*Lb. plantarum PTCC 1896, Lb. sakei DSM 20017, Lb. rhamnosus DSM 20021,* and *Lb. delbrueckii DSM 20081*) and commercial yeast (*S. cerevisiae*) contain lower acrylamide levels than bread made with yeast alone [36]. This reduction is linked to the dough’s pH, and the effectiveness varied according to the LAB strain.

#### 3.1.10. Antioxidant Activity

Whole grain flour contains a variety of phytochemicals, including antioxidants, phenolic acids, and flavonoids. Phenolic acids stand out as the most abundant antioxidants in whole grains, particularly in bran and germ. These compounds exist in various forms, such as free, soluble, conjugated, and insoluble-bound forms. Reducing the flour particle size is linked to an increased bioaccessibility of phenolic acids [60]. Traditional SD LAB starter cultures produce essential and non-essential amino acids, flavonoids, and antioxidant peptides. These compounds contribute to nutritional improvement and offer protection against oxidative stress and degenerative diseases through their phenolic content [21].

Incorporating SD fermentation has been found to improve the profile of bioactive compounds by increasing free ferulic acid. SD fermentation is known to elevate the levels of extractable phenolic compounds, primarily due to the enzymatic breakdown of cereal cell wall components [54]. This process leads to an increase in antioxidant activities [26]. LAB, particularly *Lb. plantarum* LG1034, exhibits a robust ability to enhance the total polyphenol content of SD by an impressive 82.6%. The DPPH free radical scavenging ability of SD, fermented by *Lb. plantarum* LG1034, demonstrates a remarkable strength, being 3.41 times that of the yeast-fermented one. Therefore, the antioxidant capacity of bread fermented with SD surpasses that of common white bread [26].

### 3.2. Protein Properties and Functionality Change

SD and yeast fermentation each uniquely influence protein properties and functionality throughout the baking process. Firstly, SD fermentation increases the efficiency of protein digestion and breakdown compared to yeast fermentation. SD bread is 16% more digestible, and the protein’s biological value is higher than yeast-fermented bread [33]. This digestion and breakdown of proteins take place through the synergistic effects of various organic acids (acetic acid and lactic acid), pH reduction, and enzymes produced during SD fermentation [11,61,62]. Proteins are partially broken down into smaller peptides and amino acids by proteolytic enzymes produced by the lactic acid bacteria found in SD [10,63] and become easily digestible, which increases their accessibility for absorption in the gastrointestinal tract [11]. Furthermore, the influence of SD fermentation on the complex protein network of a gluten matrix, specifically the secondary structure of the gluten protein, is different from that of yeast fermentation [34,61]. The enzymes produced by LAB during SD fermentation, such as proteases, initiate the partial breakdown of gluten in proteins and modify their secondary structure. In particular, gluten proteins experience various levels of depolymerization, resulting in the creation of distinct microstructures, such as fibrous networks and lamellar structures. This transformation is linked with the rise of β-sheet structures [11]. Each LAB strain induces distinct changes in the protein structure [61]. For example, the lamellar structures of the fermented gluten proteins in WWF by *Lb. fermentum* and *Lb. plantarum* (type II SD fermentation) are dominated by parallel β-sheet conformations [61]. The transformation of the protein matrix due to SD fermentation is reflected in the changes to the dough’s rheological characteristics [11]. Yet, the differences in protein structure and functionality between SD and yeast fermentation have not been thoroughly investigated.

### 3.3. Starch Properties and Behavior

Due to the unique fermentation conditions, wheat starch behavior in dough and bread varies between SD and yeast fermentation. SD fermentation has a more pronounced effect on starch hydrolysis, which breaks down complex starch molecules into simpler forms. The enzymes released by LAB during SD fermentation, like amylases, facilitate this starch hydrolysis [64]. Additionally, the specific LAB strain plays a role in this process. For instance, monosaccharides like glucose and fructose, along with maltose/sucrose, isomaltose, and dextrin, have been found in SD fermentations using homofermentative strains. In contrast, fermentations with heterofermentative strains only revealed maltose/sucrose, isomaltose, and dextrin [11]. Although some starch can be converted into simple sugars by yeast during fermentation, the enzymatic activity is usually much lower compared to SD.

The influence of SD fermentation on starch properties leads to an increased shelf life of the products. The shelf life of bread is commonly reduced due to a physiochemical decline known as staling, which leads to a hard and crumbly texture, diminishing the fresh-baked flavor. Following gelatinization, starch amylopectin experiences retrogradation, wherein it reverts to a more structured state. This process influences bread’s texture and its staling progression [11]. It has been reported that SD fermentation slows down starch retrogradation and delays bread staling [11]. The organic acids (lactic acid and acetic acid) produced during SD fermentation lower the pH of the dough. This acidic environment prevents the reassociation of starch molecules and retards retrogradation [11]. Additionally, LAB and their by-products, such as EPSs, help to maintain a softer crumb and delay bread staling [21]. EPSs have the ability to bind to water molecules, forming a gel-like matrix within the bread crumb [21]. This gel-like matrix can create a physical barrier around starch molecules, limiting their ability to undergo retrogradation. Moreover, water-holding properties help to retain the moisture within the bread’s crumb. This moisture retention prevents the bread from drying out too quickly, contributing to a softer texture for a longer period [21]. It is worth mentioning that the influence of SD fermentation on starch retrogradation is strongly dependent on the type and quantity of acidity produced. For instance, acetic acid has been found to have a more pronounced inhibitory effect on starch retrogradation compared to lactic acid [11]. Different SD breads with comparable acidity levels display various staling speeds. As acidity levels are determined by the fermentation environment and the LAB species used, further study should be carried out in optimizing the fermentation process to obtain the best acidic condition to increase bread quality and shelf life by delaying bread staling.

### 3.4. Volatile Compounds

**Figure 2:**
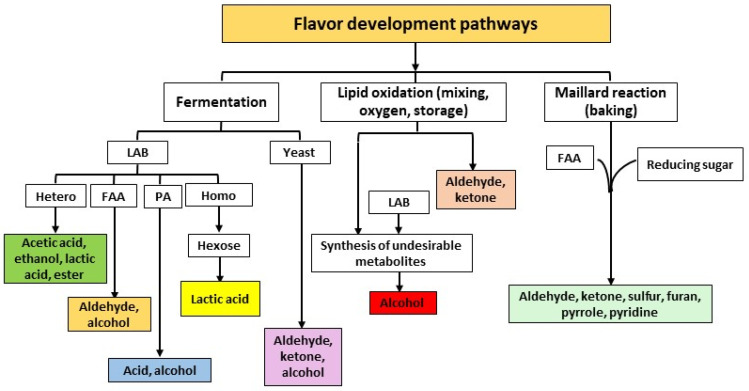
Flavor development pathways for SD and SD bread. SD: sourdough; LAB: lactic acid bacteria; FAA: free amino acid; PA: pyruvic acid.

Undoubtedly, flavor, a fusion of smell and taste, is the top factor influencing consumer preferences for baked products. While taste consists of aromatic and sapid elements, smell is generated by volatile compounds with diverse olfactory characteristics. SD products are renowned for their enriched aroma and unique flavor, setting them apart from yeast-based items. Compared to yeast fermentation, SD fermentation generates a higher count of both volatile and non-volatile molecules. The steps of dough mixing, fermentation, baking, and fat oxidation play a significant role in shaping the volatile composition of SD bread (Figure 2) [4,37].

Fermentation is the central mechanism for producing volatiles in SD, primarily by yielding acids, alcohols, aldehydes, esters, and ketones through the collective activity of yeast and LAB [65]. Competition between yeast and LAB impacts the production of the volatile compounds in SD bread. The metabolic and kinetic processes in yeast and LAB fermentation are unique, leading to the production of specific volatile compounds. LAB generates these compounds by fermenting the carbohydrates in the dough, yielding organic acids, alcohol, and various other metabolites. Furthermore, LAB promotes proteolytic activity, which breaks down proteins during SD fermentation, producing free amino acids. These amino acids then either degrade into aldehydes or transform into the respective alcohols [37]. In addition, free amino acids can act as aroma precursors as they take part in the Maillard reaction and produce volatile compounds in bread [37].

Certain flavoring compounds may result from the oxidation of fats, a process influenced by the presence of active enzymes like lipooxygenase, especially during aerobic fermentation in the mixing and storage stages of flour [4]. This oxidation of fats can occasionally create undesirable metabolites. Typically, the fermentation process serves to mitigate the adverse effects of lipid oxidation since certain LAB have the ability to convert undesirable compounds into related alcohols. Moreover, some of these components transform into volatile compounds that are lost during the baking process. The primary products of fat oxidation in bakery items consist of aldehydes and ketones. On the other hand, yeasts have volatile compounds through the fermentation of sugars, producing alcohol, esters, and other aroma-active compounds [37].

In SD and SD bread, 196 volatile compounds have been identified, including 43 aldehydes, 35 alcohols, 33 esters, 19 ketones, 14 acids, 13 furans, 11 pyrazines, 2 lactones, 2 sulfurs, 21 other compounds, and alkanes [37]. According to another study, 102 compounds were tentatively identified, including acids (10), alcohols (34), aldehydes (16), esters (15), furans (5), ketones (7), lactones (4), sulfur compounds (2), and hydrocarbons (9) [38]. Table 5 represents the principal volatile compounds present in SD and SD bread with their respective odor type and concentration.

As fermentation continues, the volatile composition changes. Yeasts are dominant during the initial three hours of SD fermentation, followed by iso-alcohols [4]. There is a surge in the total volatile content between the fifth and ninth hours of fermentation, which then remains stable for 24 h. After the 24 h mark, the volatile content diminishes [4]. Therefore, to achieve the desired flavor profile, the fermentation time needs to be adjusted.

### 3.5. Negative Aspects of SD-Fermented Bread

Although there are lots of positive things involved in SD-fermented bread, it presents several challenges, including economic, technological, and process control aspects. The longer fermentation time and the need for maintaining an SD starter can increase production costs and labor requirements compared to yeast bread. However, due to the breakdown of the gluten protein in SD fermentation [11], SD bread may have a lower specific volume, making it less airy and more compact, which can result in a firmer crumb texture compared to yeast-leavened bread. SD baking involves complex microbiological interactions that can be difficult to manage, resulting in inconsistent bread quality. The prolonged and variable fermentation process requires precise monitoring and control, making it less predictable and scalable for large-scale commercial baking. Additionally, the denser texture and distinctive tangy flavor of SD may not appeal to all consumers, potentially limiting its marketability. These factors collectively make SD bread production more demanding and less cost-effective than conventional yeast bread.

## 4. Factors Influencing the Quality of SD and SD Bread

### 4.1. Fermentation Process: Time, Temperature, and SD Type

The fermentation setting or condition is pivotal in determining the quality of SD and its end products. Notably, fermentation duration is a key factor, as it establishes the acidity level, overseeing a multitude of biochemical reactions that influence the final product’s quality. Extended fermentation periods lead to reduced pH values in both SD and SD bread [68]. For example, after 4 h of fermentation (at 30 °C), SD typically exhibits pH values between 4.51 and 4.73, while SD bread falls between 4.45 and 4.74. However, with a fermentation duration of 10 h, the pH values for SD range from 3.30 to 3.50 and for SD breads, it is between 3.20 and 3.40, contingent on the technique and starter culture used. In general, for wheat sourdoughs, it typically falls between 3.5 and 4.3 [68,69]. The decrease in pH induced by SD notably influences the dough’s rheological properties and the overall quality of the bread. Significant changes include a general decrease in elasticity and greater extensibility of dough and hardness of bread [34]. At pH levels between 3.8 and 4.1, the increase in the extensibility of the dough and the hardness of the bread is most noticeable. Accordingly, the fermentation time and pH influence the bread volume. For example, bread fermented for 6 h shows the highest specific volumes. Following that, as the pH declines, the specific volume also decreases [68,69].

On the other hand, the degradation of the gluten protein is directly proportional to the SD fermentation time as it decreases pH [33]. At pH values of ≤4.0, wheat proteinases that break down gluten proteins function best. The proteolytic degradation of glutenin subunits first happens after 6 h of fermentation and becomes more pronounced after 24 h [4]. The extent of gluten protein hydrolysis is contingent on the degree of acidification, which varies with fermentation duration, significantly impacting the flavor profile as well [70]. SD’s acidification intensity also plays a role in delaying bread staling [71]. Thus, pH can be deemed a critical factor in determining the quality of SD bread. While a decreased pH through extended fermentation enhances the bread’s aroma, it might adversely affect the bread’s texture and the dough’s rheological properties due to the proteolytic breakdown of wheat flour proteins. Therefore, fine-tuning the fermentation duration to regulate pH is of utmost importance.

The temperature during fermentation is another significant factor affecting the quality of SD and its end product. Variations in the fermentation temperature account for approximately 44.10% of the differences observed between different SD [34]. The hardness of SD bread is greatly influenced by the fermentation temperature. In general, a rise in the fermentation temperature decreases the hardness of SD bread and improves the bread quality. For example, fermentation at 35 °C leads to less hard bread compared to fermentation that follows 28 °C temperature [32,34,69]. Moreover, despite the lack of statistical significance, the fermentation temperature demonstrates an impact on the springiness, cohesiveness, and resilience values of SD [32]. Furthermore, the fermentation temperature can influence the elastic properties of SD. At lower fermentation temperatures (28 °C), SD tends to display a more pronounced elastic behavior, indicated by a higher elastic modulus in the dough.

Fermentation temperature also plays a role in determining the types of volatile compounds produced during the process. At higher fermentation temperatures (35 °C), there is a noticeable increase in aldehydes and esters, while fewer alcohols are produced compared to lower temperatures (28 °C) [34]. The fermentation quotient (FQ), defined as the molar ratio of lactic acid to acetic acid, is a vital metric for evaluating the effectiveness of the SD fermentation process. Elevated fermentation temperatures can disrupt the FQ balance by leading to increased lactic acid production and decreased acetic acid values [34,72]. In contrast, at lower or optimum temperatures, lactic acid production decreases while acetic acid increases. Consequently, this leads to a significant reduction in FQ values, keeping them within the optimal range of 2.0–2.7 [34,72]. Fermentation temperature also has an impact on SD’s pH reduction. At high fermentation temperatures, pH reduces faster than at low temperatures. For example, it takes 11 h at 25 °C and 9 h at 35 °C to reach the ideal pH required to produce SD [69]. Thus, to achieve top-quality SD bread, it is essential to optimize the fermentation temperature.

The type of SD fermentation also affects the quality of sourdough and sourdough bread significantly. For example, bread from type II SD fermentation boasts a larger specific volume (41 to 46% higher) compared to that of type I [32]. This is because the pure cultures used in the type II process lead to a more pronounced volume increase in bread than the spontaneous fermentation seen in type I. Conversely, type I SD bread exhibits significantly greater chewiness (3 to 25% and 117 to 133% higher in WWF and refined flour SD bread, respectively) and hardness (62 to 80% and 160 to 270% higher in WWF and refined flour SD bread, respectively) compared to type II [32,68]. SD fermentation type also has a noteworthy effect on crust color for both refined flour and WWF products [69]. For instance, type I fermentation can yield a higher L* value compared to type II fermentation for both refined (up to 20%) and WWF products (up to 30%) [32]. Notably, the SD fermentation type has a significant effect on the a* value for WWF products based on the fermentation temperature but does not have a significant effect on refined flour products [32,69]. For example, the temperature does not significantly affect the a* value in type II fermentation. Conversely, in type I fermentation, the a* value is notably higher at 25 °C compared to 30 °C. However, for both refined and WWF products, fermentation type has a significant effect on the b* value when focusing on fermentation temperature [32]. For instance, a higher b* value is observed at 25 °C in type I fermentation and at 30 °C in type II fermentation. On the other hand, the fermentation type and temperature do not have any significant impact on the crumb color of wheat bread. SD type also greatly impacts pH, enzyme activity, protein and starch digestion, dough rheology, flavor, and bread’s physical properties, which have been discussed previously.

### 4.2. Flour Particle Size

Generally, the particle size of wheat flour plays a crucial role in the fermentation process, dough rheological characteristics, and the final baked product, as extensively documented in yeast-fermented breads. Even with limited studies focusing on the effect of particle size on SD quality, it is evident that it is a major determinant in the quality of SD products. Flour particle size strongly influences the functional attributes of starch and protein, both refined and WWF. For instance, the degree of starch damage increases as flour particle size decreases [73]. Damaged starch significantly influences dough’s physical characteristics. For example, an overly high concentration of damaged starch (above 12%) can lead to increased water absorption, resulting in dough that is softer and stickier. Such dough struggles to sustain its expansion during proofing, which often results in bread with reduced volume [74]. Starch viscosity, as determined using the rapid visco analyzer (RVA), is closely related to the final quality of the bread and can also predict the bread’s firming behavior [75]. When the particle size is reduced, viscosity tends to increase. This rise in viscosity is attributed to the damaged starch granules formed during the reduction process. Damaged granules are more efficient at absorbing water and swell more readily, releasing amylose and amylopectin more easily, leading to increased starch paste viscosity [76,77]. Particle size reduction also influences bread firmness by changing the amylose/amylopectin ratio [78].

Variations in particle size can also impact protein functionalities. Although the total protein content remains consistent despite reductions in particle size, tests like glutopeak indicate enhanced gluten strength and wet gluten analyses demonstrate improved gluten aggregation capability [79]. The combination of heightened gluten strength and aggregation ability contributes to an increased specific volume in bread [77].

In particular, flour particle size plays a pivotal role in determining the physical properties of WWF dough, such as dough strength and extensibility. This is due to the coarse nature of the bran present in WWF, which typically disrupts the functionality of starch and proteins. As the particle size of WWF reduces, there is a noticeable increase in stability time, dough development time (DDT), and time to break down, all indicative of enhanced dough strength [77]. Dough made from finer WWF showcases the lowest mixing tolerance index (MTI) value in comparison to dough made from coarser WWF, indicating an improvement and resulting in better quality dough [77] because effective water absorption capacity and gluten network formation due to particle size reduction contribute to dough’s stability and uniformity, reducing the likelihood of over-mixing or under-mixing [80]. The reduction in particle size also contributes to the increasing extensibility and elasticity of the dough [77]. The presence of more bran particles can hinder the proteins in the WWF from coming together, leading to a less robust gluten structure [81].

The color of WWF and the finished product can be improved significantly by reducing the particle size [82]. Additionally, reducing the particle size of wheat bran can effectively diminish its phytic acid content, leading to fewer anti-nutritional components [82]. Remarkably, the synergy of particle size reduction and SD fermentation appears to be more potent in decreasing phytic acid content than using either method independently. Phytic acid concentrations decrease by 12.4–56.9% with particle size reduction only, but when combined with fermentation, the reduction ranges from 28.4 to 57.3% [82]. However, particle size reduction has detrimental impacts on the amount of total, soluble, and insoluble dietary fiber.

### 4.3. Protein Content and Quality

Protein content and quality are key factors in determining the characteristics of dough and the eventual quality of bread. While measuring protein content is straightforward, assessing protein quality is more complicated, as it encompasses many protein biochemical properties. Broadly, protein quality can be delineated by the relative distribution of various gluten protein classes, which primarily influence dough’s rheological attributes [83]. Gluten proteins can be categorized into two primary classes: glutenins and gliadins. Glutenins comprise high molecular weight (HMW) and low molecular weight (LMW) subunits. These subunits form polymeric proteins through intermolecular disulfide bonds. Conversely, gliadin proteins, with their subgroups α, β, ꞷ, and Ƴ, are monomeric and do not form disulfide bridges between their polypeptide chains [84]. The abundance of these protein classes can vary among different wheat types and cultivars. Consequently, the balance of these protein classes plays a crucial role in determining bread quality, with a specific ratio being crucial to achieving the desired bread characteristics.

While protein content, especially gluten content, is crucial for wheat products, its impact on SD is even more pronounced. SD bread preparation involves a longer fermentation process compared to yeast fermentation, leading to more common gluten degradation. Due to this prolonged fermentation, wheat gluten structures in SD may affect dough characteristics. Therefore, using high-protein flour (10–14%), which is rich in gluten, can result in better dough development. Such a high protein content in whole wheat flour establishes a resilient gluten network capable of withstanding the extended fermentation periods (12 to 24 h) typical of SD [70]. Compared to SD bread made from low-protein flour, bread made from high-protein flour exhibits increased moisture content, better porosity, and a larger specific volume [85].

Additionally, SD crafted with high-protein WWF produces more lactic and acetic acids than those made with low-protein flour. This leads to a more acidic environment with a lower pH, conditions that are optimal for SD fermentation and bread quality [70]. Such an acidic environment further enhances protein hydrolysis, resulting in the formation of amino acids and small peptides. This not only aids in protein digestibility but also in the generation of volatile compounds that enrich flavor. To augment the flavor of SD bread, a heightened proteolysis process is employed during its fermentation period (12 to 24 h) [4]. Furthermore, a higher protein concentration in flour positively impacts the bread’s L* and a* color attributes, although it does not significantly affect the b* value [85]. High-protein SD bread garners superior sensory ratings due to its softer texture, enhanced porosity, appealing appearance, and richer flavor when compared to its low-protein counterpart.

While extensive research on the role of protein quality in SD bread is still lacking, its importance in yeast-fermented bread is well-established. Specifically, the balance between monomeric gliadin and polymeric glutenin, which together form the gluten complex, is crucial. Gliadin primarily influences dough viscosity and extensibility, while glutenin contributes to dough strength and elasticity [86]. The quality of flour hinges on a delicate equilibrium between gliadin and glutenin. For optimal bread baking, a certain harmony between the dough’s viscosity and its elasticity/strength is essential. For instance, a bread loaf with reduced volume typically signifies the presence of insufficiently elastic gluten. While enhanced elasticity can increase bread volume, an overly elastic gluten matrix can decrease the expansion of gas cells, leading to decreased loaf volume. Hence, even with limited direct evidence, it is evident that protein quality plays a significant role in determining SD’s functional properties and the final bread quality. While some studies have shed light on the significance of protein content for SD bread and its effects on its quality, there is limited information regarding the specific influence of protein composition, particularly on the relative proportion of protein classes.

### 4.4. Starch Properties and Composition

As the predominant component of flour, starch significantly influences dough’s rheological properties and the quality of the final product. Wheat starch consists of amylose and amylopectin, each with distinct biochemical and molecular compositions [87]. The amylose-to-amylopectin ratio is the primary physicochemical factor that determines its appropriateness for specific applications. Additionally, the size distribution of starch granules also impacts dough characteristics [87]. The characteristics of starch affect its behavior, including its swelling, gelatinization, pasting, and retrogradation properties.

During gelatinization, starch granules swell, absorb water and disrupt their internal crystalline structures. This leads to granule breakdown [87]. Subsequent to gelatinization is the pasting process, characterized by further granule swelling and the leaching of amylose molecules, culminating in the formation of a viscous gel. The point at which this viscosity is maximized as temperature increases is termed peak viscosity. This metric provides insight into the starch’s water retention capability and often correlates with the quality attributes of the sample [87].

Although the impact of starch properties on its modifications in SD bread has not been extensively studied, its significance in yeast-fermented bread is well-documented. Wheat starches show decreasing breakdown and peak viscosities with rising total amylose content as part of their pasting properties. So, higher peak viscosity with low amylose content indicates a soft end product since the high amylose content generally causes hardness [78,87]. Higher breakdown viscosity indicates easy cooking, which shows that flour containing starch with a high breakdown viscosity and lower amylose concentration will make baking easier [75]. Setback viscosity (measured using an RVA) to measure starch pasting properties is related to bread staling [75,87]. Low setback viscosities suggest slower starch retrogradation, which in turn indicates a reduced likelihood of bread staling, ultimately extending the bread’s shelf life [11]. The characteristics of starch, including its amylose content, gelatinization, and pasting properties, undeniably influence the quality of yeast-fermented bread. However, comprehensive research in this area on SD-fermented baking is currently limited.

### 4.5. Dietary Fiber Composition

Dietary fiber plays a crucial role in determining the quality of SD bread. SD bread crafted from whole wheat flour (WWF) boasts a higher dietary fiber content compared to its refined flour counterpart [11]. Broadly, the major elements of wheat dietary fiber, such as insoluble dietary fiber, water unextractable AXs, and bran granules in WWF, have a negative impact on dough’s physical attributes [11]. However, WWF quality is improved when there is an upsurge in soluble dietary fiber, which notably elevates during SD fermentation [11]. Soluble dietary fiber possesses the ability to absorb water, enhancing the bread’s moisture retention capability and making the crumb softer and more tender. This prevents the bread from drying out or becoming stale [11].

AXs (one of the key components of dietary fiber) are two types: water-extractable arabinoxylan (WEAX) and water-unextractable arabinoxylan (WUAX) [88]. Among them, WEAX makes a positive contribution to the quality of SD bread [11]. For instance, although it does not establish a definitive connection, the content of WEAX has a positive correlation with the specific volume of SD bread. Introducing WEAX during the dough mixing process typically enhances dough consistency and its ability to absorb water. Moreover, the presence of WEAX during baking can bolster the stability of gas cells and amplify the dough’s gas retention capability. This results in a prolonged oven spring and improves various bread quality attributes such as specific volume (up to 25%), crumb structure (reduced pore diameter of up to 3.5%), firmness (reduction of up to 77%), and overall texture [11,89]. WUAX can lead to dough degradation through a variety of mechanisms: (i) contributing to the dilution of gluten proteins and starch; (ii) competing with proteins for water during gluten network formation, leading to the inadequate hydration of both gluten proteins and starch; (iii) Acting as physical barriers to gluten network formation, known as the spatial barrier effect; and (iv) dough containing these arabinoxylans exhibits a firmer texture, decreased recovery and viscoelasticity, and diminished tensile strength; its transformation into the water-extractable form via the activation of xylanase during SD fermentation positively impacts the quality of the final products [11]. While numerous studies have explored the nutritional effects of dietary fiber on SD bread, there is a notable gap in the research regarding how dietary fiber influences the fermentation process, dough’s physical properties, and the bread quality of SD.

## 5. Application of SD Technology to Unconventional Cereal Flour and Non-Flour Ingredients

SD bread is typically made with wheat flour, but alternative non-conventional flour such as rye, barley, quinoa, triticale, sorghum, oat, maize, intermediate wheatgrass (IWG), and ancient (emmer, spelt, and Khorasan) flour can also be used [59]. These alternatives can enhance the bread’s quality and cater to consumer preferences. Moreover, using non-conventional flour in SD can boost the health benefits of baked goods. SD made from a blend of chickpea, faba bean, amaranth, buckwheat, and quinoa flour produces ten times more gamma-aminobutyric acid (GABA) compared to SD made from wheat flour [59]. GABA, a non-protein amino acid, acts as an inhibitory neurotransmitter in the central nervous system and offers health benefits such as managing hypertension, hyperglycemia, and inflammation, protecting tissues from degeneration, and serving as an antioxidant [90].

IWG, a perennial wheat, offers significant environmental benefits over annual plants. Nutritionally, IWG surpasses wheat with 56.92% more protein, 57.89% more fiber, and substantially higher mineral content, including potassium (78.37%), calcium (131.57%), magnesium (20.70%), phosphorus (52.87%), iron (84.35%), manganese (31.80%), and zinc (48%) [91]. Despite these advantages, its primary drawback is the reduced presence of high molecular weight glutenin subunits (HMW-GSs), which leads to poor performance in traditional bread products [91]. However, IWG’s high protein, ash, and starch content make it a promising candidate for SD fermentation. IWG SD shows higher FQ (44% higher) and lactic acid production, corresponding with a high enumeration of LAB. Although IWG SD bread is more acidic, this does not negatively affect its quality, including volume and post bake firmness. Sensory analyses using the just about right (JAR) scale indicate that IWG SD bread receives an average score above three, meeting consumer expectations [91]. With formula optimization, IWG SD bread samples can achieve greater alignment with consumer preferences, highlighting the potential of SD technology to produce highly nutritious SD bread using IWG.

SD from ancient (emmer, spelt, and Khorasan) and modern wheat differently affect dough viscoelastic properties, bread volume, texture, firming rate, color, and sensory properties, but not water activity. Khorasan and emmer sourdoughs produce bread with low volume and hard texture due to their gluten properties, while spelt and modern wheat sourdoughs yield bread with similar volume and texture [92]. SD breads from ancient wheat have a milder sour taste, odor, and flavor, enhancing their sensory appeal [92].

Grain legumes, rich in protein, fiber, minerals, and bioactive compounds, have been extensively studied to enhance the nutritional profile of foods [93]. Fortifying cereals with legume flour is recognized as an effective strategy to improve the nutritional quality of cereal-based foods, expanding technological and market opportunities for products like bread, bakery items, and pasta [93]. For example, the faba bean, with its high protein content (30%) and numerous health benefits, is widely utilized in food [94]. Substituting wheat flour with faba bean flour significantly affects composite bread’s properties. Replacing 30% of wheat flour with faba bean flour, whether native or fermented, reveals notable structural and nutritional differences. Native faba bean flour slightly reduces bread volume and increases hardness due to its impact on gluten formation and gas retention [94]. Fermentation mitigates these issues, preserving crumb porosity similar to wheat bread by modifying the flour’s physical and chemical properties. Nutritionally, faba bean flour enhances the bread’s protein content from 11.6% to 16.5%. While native faba bean flour does not alter protein digestibility (64%), the SD variant improves it to 73%, breaking down anti-nutritional factors and modifying protein structures [94]. Faba bean SD bread shows superior nutritional indexes, including higher free amino acid profiles (377.85% higher than wheat bread), essential amino acid indexes (9.89% higher), improved protein chemical scores (44.86% higher), and higher biological value indexes (13.6% higher), indicating better essential amino acid balance and overall protein quality [94]. This makes the SD variant more nutritious and beneficial. Additionally, adding liquid or freeze-dried chickpea SD to wheat bread recipes can improve the specific volume, texture, and sensory qualities of the loaves, as well as extend their shelf life [95].

Additionally, various non-flour ingredients commonly used in the kitchen, such as fruits, herbs, honey, milk, salt, sugar, and yogurt, can be incorporated into the flour–water mixture to produce SD [14]. Some of these ingredients introduce specific microorganisms, serving as an additional microbial inoculum [14]. Others provide substrates or co-substrates for microbial fermentation and conversion, thereby activating or selecting microorganisms naturally present in either the flour or the added ingredients. For example, incorporating apple juice with wheat flour boosts lactic acid production and increases the population of LAB [96]; adding honey to wheat and rye flour influences SD bread dough development [97]; applying lemon juice with wheat flour enhances the production of acetic acid, lactic acid, acetoin, and diacetyl [96]. Therefore, using an appropriate ingredient in SD production can impact both the microbial composition and the organoleptic properties of SD bread, particularly its aroma and taste profiles [98].

Despite the promising advancements in SD bread research, including the use of ingredients such as IWG, ancient flour, faba bean flour, chickpea, and various kitchen staples to enhance nutritional profiles and microbial activity, current studies remain insufficient. Further comprehensive research is essential to fully understand and optimize these innovations, ensuring they meet consumer expectations and maximize the nutritional and sensory benefits of sourdough bread.

## 6. Potential Usage/Market of Frozen SD

As highlighted in the previous sections, SD has captured a significant share in various global markets, prominently featuring in traditional baked goods like pizza bases, cakes, pastries, bread, and buns. However, the landscape of the wheat-based bakery industry has undergone a remarkable transformation over recent years, driven primarily by technological advancements and evolving consumer preferences. Notably, the frozen dough sector is witnessing rapid growth and now commands a sizable part of the bakery market. As lifestyles change, the demand for frozen dough products has surged. As per the report released by Future Market Insights [99], the global frozen dough market is estimated to reach a valuation of USD 17.5 billion in 2024. It is projected to surge to USD 29.5 billion by 2034, marking a CAGR of 5.4% from 2024 to 2034. However, this booming sector is heavily dominated by yeast-based fermentation, leaving SD enthusiasts missing out on the benefits of this expanding area. There is a pressing need for in-depth research to tailor SD methodologies to the requirements of the frozen goods industry.

Baker’s yeast is the primary leavening agent used in frozen dough technology, and extra additions are used to compensate for the dough’s poor gas retention and baking performance [100]. Previously, SD made with *Lb. plantarum* and a variant of *S. cerevisiae* could be preserved for up to two weeks. However, the finished product was provided with an inadequate rating, and refreshing frozen SD is necessary to obtain the best quality [100]. The particular application of SD in conjunction with freezing could be classed as pre-fermented frozen dough [15]. Four common techniques for preserving SD include back-slopping, drying, refrigeration, and freezing. Refrigeration and drying methods maintain their effectiveness for about 30 days. In contrast, freezing can partially preserve the initial LAB for up to 90 days [15,101]. While the acidification ability of reactivated SD remains consistent, their leavening capability falls short compared to fresh SD. This happens because, during the freezing process, the development of ice crystals and the increase in intracellular salt concentration below the freezing point can prompt LAB cells to experience water leakage, ultimately resulting in a reduction in cell viability [15]. However, inulin falls within the category of carbohydrates known as fructans and is considered a potential cryoprotectant due to its ability to preserve protein structure; it is helpful in protecting the viable cell of LAB and their acid-producing capacity during freezing [15]. In addition, when SD is utilized as frozen dough alongside honey, cryoprotectants (glucose, sucrose, inulin, etc.), or both, its leavening efficiency is enhanced compared to using SD by itself [15,102].

Compared to bread crafted from fresh or traditional frozen dough, those made with pre-fermented frozen dough enriched with honey, fructose, glucose, or a combination of honey and cryoprotectant yield softer loaves. Breads that incorporate honey are notably softer, and the inclusion of standard additives allows them to achieve a volume comparable to bread made from unfrozen dough, regardless of whether SD is used [102]. This illustrates the potential for industrial bakeries to utilize frozen SD effectively. Nonetheless, there is a need for more in-depth research into the capabilities of SD in the frozen market, and the findings from such studies should be shared and implemented at an industrial scale.

## 7. Conclusions

SD fermentation enhances the quality of bread by improving protein and starch functionality, increasing dietary fiber, boosting postbiotic and antioxidant activity, and reducing ANFs and FODMAPs, which meet the growing consumer demand for healthier bread options. This fermentation process allows for product differentiation, creating unique and specialized bread varieties that cater to health-conscious consumers and those with dietary restrictions. Incorporating alternative grains and non-flour ingredients, such as fruits, herbs, and dairy products, offers innovation in product development and aligns with consumer preferences for novel and nutritious baked goods. Understanding the factors that influence sourdough quality, such as fermentation environment and flour particle size, helps bakeries optimize their processes for consistent, high-quality production. The expanding market for SD bread presents commercial opportunities, including the potential for frozen SD products, which offer convenience and extended shelf life, helping bakeries manage inventory and reduce waste. Educating consumers about the health benefits of SD and leveraging its sustainability through alternative grains can enhance market demand and support branding efforts. Thus, SD fermentation holds the potential to revolutionize commercial baking by aligning with health trends, enabling product innovation, and expanding market reach.

## Figures and Tables

**Table 1:** Commonly identified LAB and yeast in SD; adopted from [4,5,6,7].

Obligately Heterofermentative	Facultatively Heterofermentative	Obligately Homofermentative	Yeast
*Lb. acidifarinae*	*Lb. alimentarius*	*Lb. acidophilus*	*S. cerevisiae*
*Lb. brevis*	*Lb. buchneri*	*Lb. amylolyticus*	*S. bayanus*
*Lb. buchneri*	*Lc. Lactis*	*Lb. amylophilus*	*K. exigua*
*Lb. cellobiosus*	*Lb. paracasei*	*Lb. amylovorus*	*K. humilis*
*Lb. crustorum*	*Lb. kimchi*	*Lb. bulgaricus*	*K. servazzi*
*Lb. curvatus*	*Lb. paralimentarius*	*Lb. farciminsis*	*K. exigua*
*Lb. fermentum*	*Lb. pentosus*	*Lb. johnsonii*	*Pi. kudriavzevii*
*Lb. frumenti*	*Lb. plantarum*	*Le. lactis*	*T. delbrueckii*
*Lb. fructivorans*	*Lb. sakei*	*Lb. heilongjiangensis*	*Wi. anomalus*
*Lb. hammesii*	*Lb. casei*	*Lb. crustorum*	*Pi. kudriavzevii*
*Lb. hilgardii*	*Lb. rhamnosus*	*Lb. amylovorus*	*C. tropicalis*
*Lb. homohiochi*	*Lb. xianfangensis*	*Lb. crispatus*	*C. glabrata*
*Lb. namurensis*	*Le. holzapfelii*	*Lb. delbrueckii*	*C. krusei*
*Lb. nantensis*	*P. acidilactici*	*Lb. reuteri*	*C. pelicullosa*
*Lb. panis*	*P. pentosaceus*	*Lb. nodensis*	*Y. keelungensis*
*Lb. reuteri*		*Lb. helveticus*	*T. delbrueckii*
*Lb. parabuchneri*		*Lb. salivarius*	*R. mucilaginosa*
*Lb. rossiae*		*Lb. gallinarum*	
*Lb. sanfranciscensis*		*Lb. mindensis*	
*Lb. secaliphilus*		*E. durans*	
*Lb. siliginis*		*E. faecalis*	
*Lb. spicheri*		*E. faecium*	
*Lb. zymae*		*P. parvulus*	
*Le. citreum*			
*Le. gelidum*			
*Le. mesenteroides*			
*W. cibaria*			
*W. confuse*			
*W. viridescens*			

E., Enterococcus, Lb., Lactobacillus, Lc., Lactococcus, Le., Leuconostoc, P., Pediococcus, W., Weissella, S., Saccharomyces, K., Kazachstania, Pi., Pichia, T., Torulaspora, Wi., Wickerhamomyces, C., Candida, Y., Yarrowia, R., Rhodotorula.

**Table 2:** SD market in different nations along with the flour used to make SD products; adopted from [18,19,20].

Flour Type	Application of SD	Country of Commercialization
Wheat	Bread	Italy, Germany, Argentina, Spain, France and Brazil (in Brazil partnership with Vallens), France, Belgium Mexico, Spain, Morocco, and Brazil
	Bread and pizza	France and France and Brazil (in Brazil’s partnership with Vallens)
	Bakery products in general	USA, France, Italy, Germany, Belgium, France and Brazil (in Brazil partnership with Vallens), France, Belgium, Mexico, Spain, Morocco, and Brazil
	French bread; San Francisco bread; pancake and waffle mix	USA, France, and Brazil (in Brazil’s partnership with Vallens)
	San Francisco bread and bakery products in general (culture from New Zealand, France, and Italy)	New Zealand
Whole wheat	Bakery products in general	USA, France, France, Belgium, Mexico, Spain, Morocco, and Brazil
	Bread	France and Brazil (in Brazil’s partnership with Vallens)
Rye	Bread	Germany, France, and Brazil (in Brazil’s partnership with Vallens)
	Bakery products in general (culture from New Zealand and France)	USA, New Zealand, Germany, Belgium, France, Belgium, Mexico, Spain, Morocco, and Brazil

SD: sourdough.

**Table 3:** SD fermentation compared to yeast fermentation.

Aspect	Yeast Fermentation	SD Fermentation	Reference
Nutrient availability	Limited mineral bioavailability; minerals often remain inaccessible for digestion.	Enhanced mineral bioavailability; increases the bioavailability of iron by 10% and zinc by 25%. Produces more fermentable sugars like sorbitol and mannitol. Boosts vitamin B12, folate, and riboflavin.	[21,22,23,24]
Phytate content	Reduces phytic acid by up to 56%.	Can degrade phytic acid by up to 96.6%, significantly increasing mineral bioavailability.	[25,26]
Anti-Nutritional factors	Less effective at breaking down anti-nutritional factors (ANFs).	More effective; reduces raffinose, condensed tannins, trypsin inhibitors, and saponins significantly.	[25,27]
Postbiotic compounds	Does not produce significant levels of postbiotics.	Rich in postbiotics like short-chain fatty acids (SCFAs), β-glucan, and peptidoglycan, which provide anti-inflammatory and anti-tumor effects.	[6,21,28]
Pathogen inhibition	Does not produce compounds that significantly inhibit pathogens.	Produces bacteriocins and biosurfactants that inhibit pathogenic microorganisms and prevent biofilm formation.	[6,21,28]
Dietary fiber	Limited increase in dietary fiber availability.	Significantly increases dietary fiber availability and produces prebiotic components like arabinoxylan-oligosaccharides (AXOSs).	[22,27]
Resistant starch	Less effective at increasing resistant starch content.	Increases resistant starch by 89% to 120%. Low pH and LAB enzymes modify starch structure, making it more resistant to digestion.	[29,30]
Digestibility	Yeast bread has lower digestibility with slower gastric emptying and oro-cecal transit time.	SD bread has superior digestibility with higher nutritional indices, faster gastric emptying, and quicker oro-cecal transit time	[31]
	Higher levels of rapidly digestible starch (RDS)	Reduces rapidly digestible starch (RDS) by approximately half compared to yeast fermentation	[32]
	Less efficient protein digestion and breakdown.	Increases protein digestion efficiency by 16%; modifies gluten protein structure, leading to different rheological characteristics.	[33,34]
Glycemic index (GI)	Higher GI; can cause rapid spikes in blood sugar levels.	Lower GI; leads to gradual glucose absorption, quicker gastric emptying, activation of satiety hormones, and increased resistant starch.	[24]
FODMAPs	Less effective at reducing FODMAPs.	More effective; reduces FODMAPs, especially fructans, significantly. Produces mannitol from fructose and other sugars.	[35]
Acrylamide	Higher acrylamide levels due to the Maillard reaction during baking.	Lower acrylamide levels due to the low pH environment inhibiting its synthesis.	[36]
Antioxidant activity	Lower antioxidant activity.	Higher antioxidant activity; increases the levels of extractable phenolic compounds and free ferulic acid.	[26]
Starch properties	Lower impact on starch hydrolysis; faster staling due to starch retrogradation.	Higher starch hydrolysis; slows down starch retrogradation and delays bread staling. Organic acids and EPSs maintain softer crumb and moisture retention.	[11]
Volatile compounds	Produces fewer volatile compounds; primarily alcohol, esters, and some aroma-active compounds.	Produces a higher count of volatile compounds; enriched aroma and unique flavor. Generates acids, alcohols, aldehydes, esters, and ketones.	[3,4,37,38]

**Table 4:** Effect of fermentation on the FODMAP level in bread.

FODMAP-Measured Group	Fermentation Type	Level of Reduction (%)	Increase (%)	Reference
Fructans	SD	65	-	[41]
Fructans	SD	65–70	-	[42]
Fructans	SD	69–75	-	[35]
Fructans	Yeast	56		[35]
Fructans	Yeast	<50	-	[42]
Fructose + glucose	SD	69–82	-	[43]
Raffinose	SD	69	-	[35]
Nystose + stachyose	SD	0–86	-	[43]
Nystose + stachyose	Yeast	0	-	[43]
Sorbitol + mannitol	SD	-	172–1000	[35]
Sorbitol + mannitol	Yeast	-	0–67	[43]
Raffinose + kestose	SD	-	114–120	[35]
Raffinose + kestose	Yeast	-	0–133	[35]
Mannitol	SD	-	550	[35]
Mannitol	Yeast	-	0	[35]

SD: sourdough; FODMAPs: fermentable oligosaccharides, disaccharides, monosaccharides, and polyols.

**Table 5:** Principal volatile compounds in SD and SD bread with their respective odor type and concentration.

Group	Volatile Compound	Odor	Range Concentration (ppm)	Percent Threshold in Water (ppm)	References
Aldehydes	Hexanal	Fresh, green, fatty, aldehydic, grass, leafy, fruity, sweaty	0.00–0.14	0.0045–0.005	[2,66]
	Heptanal	Fatty, rancid, citrus, malty, aldehydic, grass, fresh, green, ozone	0.00–0.03	0.003	[20,37,67]
	Octanal	Fatty, aldehydic	-	0.0007	[37,66]
	Acetaldehyde	Pungent, aldehydic, floral, fruity	0.00–0.49	0.015–0.12	[2,3]
	2-Methylbutanal	Musty, cocoa, coffee, nut, malty, fruity, sweet, roasted	-	0.001	[38]
	Benzaldehyde	Almond, strong, sharp, sweet, bitter, cherry	0.00–0.26	0.35–3.5	[37,66]
	Nonanal	Aldehydic, rose, waxy, citrus, orange, floral	0.00–0.34	0.001	[20]
	3-Methylbutanal	Ethereal, aldehydic, chocolate, Peach, fatty, sour, roasted bread, fruity, fermented, corn flakes	-	0.0002–0.002	[37,38]
	2-Nonenal	Fatty, green, aldehydic, citrus, waxy	0.00–0.34	0.00008–0.0001	[66]
Alcohols	Ethanol	Strong, alcohol, ethereal, medicinal	0.00–15.70	100.00	[20,67]
	Isobutyl alcohol	Alcoholic	0.00–5.75	-	[20]
	1-Hexanol	Herbal, ethereal, oil, alcohol, green, fruity, sweet, woody, floral	0.00–1.04	2.50	[67]
	1-Nonanol	Floral, rose, orange, fresh, clean, fatty, oily	-	0.05	[2,3,37]
	1-Heptanol	Green, musty, leaf, woody, peony, violet, grass, sweet	-	0.003	[20,37]
	1-Octanol	Waxy, green, orange, aldehydic, rose, mushroom, citrus	0.01–0.72	0.11–0.13	[67]
	1-Pentanol	Oil, sweet, balsamine, chemical mint	0.05–0.37	4.00	[66]
	Phenylethyl alcohol	Floral	0.00–0.30	-	[2,3]
	Phenol	Phenol, plastic, rubber	-	-	[20]
	3-Methyl-1-butanol	Balsamic, alcoholic, malty	0.33–38.83	-	[66]
Esters	Methyl acetate	Ethereal, sweet, fruity, solvent, wine, cognac, rum	-	-	[20]
	Ethyl acetate	Ethereal	0.00–23.35		[20]
	Ethyl lactate	Fruity, butter, caramel, green	0.01–1.72	14.00	[37,66]
	Ethyl octanoate	Sweet, soap, fresh, fruity, wine, Waxy, apricot, banana	0.00–0.44	-	[66]
	Octyl acetate	Green, earthy, mushroom, herbal, waxy	-		[20]
Ketones	3-Hydroxy-2-butanone (acetoin)	Caramel, butter, yogurt, cream	0.02–1.42	-	[20,66]
	2,3Butanedione	Butter, caramel	0.00–0.81	-	[67]
	2-Pentanone	Sweet, fruity, ethereal, wine, banana, woody	-	70.00	[37,38]
	2-Octanone	Earthy, grass, woody, soap	-	0.05	[38]
Acids	Formic acid	Acrid, vinegar, formyl	-	450.00	[37]
	Lactic acid	Slight, not unpleasant odor	0.00–0.03		[20]
	Acetic acid	Sour, acid, pungent, sharp Vinegar	0.00–3.2		[2,3,66]
	Hexanoic acid	Fatty, sour, sweaty, cheesy	0.00–0.02	3.00	[66]
	Heptanoic acid	Cheese, fatty, sweaty	-		[20]
	Octanoic acid	Octanoic acid, cheese, fatty, sweaty, soapy, waxy, vegetable	-	3.00	[20]
	Isobutyric acid	Sweaty, butter, fatty, sour, rancid	-		[20]
	Isovaleric acid	Sweaty			[20]
	Benzoic acid	Faint balsam, urine	-	-	[38]
	Propanoic acid	Cheesy, acidic, vinegar, acrid, rancid	-	20.00	[38]
	Butanoic acid	Acetic, butter, fruity, sweet, sour, sharp	-	0.24	[38]
	Pentanoic acid	Acidic, sweat, rancid, stinky, putrid	-	3.00	[38]
Furans	2-Acetylfuran	Sweet, balsamine, almond, cocoa, caramel, coffee, burnt	-	10.00	[37,38]
	Furfural	Almond, bread-like, soil, burnt roasted, sweet, toasted, rancid	0.00–0.19	3.00–23.00	[67]
	2-Penthylfuran	Fruity, mushroom, raw nuts, Butter, green bean, floral, earthy	-		[20]
Alkanes	Limonene	Citrus	0.00–0.32	-	[66]
	Decane	Alkane	-	-	[20]

## Data Availability

The original contributions presented in the study are included in the article, further inquiries can be directed to the corresponding author.
